# Effectiveness of a lifestyle modification programme in the treatment of depression symptoms in primary care

**DOI:** 10.3389/fmed.2022.954644

**Published:** 2022-07-26

**Authors:** Alejandra Aguilar-Latorre, Guillermo Pérez Algorta, Capilla Navarro-Guzmán, María J. Serrano-Ripoll, Bárbara Oliván-Blázquez

**Affiliations:** ^1^Primary Healthcare Center Arrabal, Institute for Health Research Aragón (IIS Aragón), Zaragoza, Spain; ^2^Division of Health Research, Faculty of Health and Medicine, Lancaster University, Lancaster, United Kingdom; ^3^Department of Psychology, University of the Balearic Islands, Palma, Spain; ^4^Primary Care Research Unit of Mallorca, Balearic Islands Health Services, Palma, Spain; ^5^Research in Preventive Activities and Promotion and in Cancer Illes Balears (GRAPP-CAIB), Balearic Islands Health Research Institute (IdISBa), Palma, Spain; ^6^Research Network on Chronicity, Primary Care and Health Promotion (RICAPPS), Barcelona, Spain; ^7^Department of Psychology and Sociology, University of Zaragoza, Zaragoza, Spain

**Keywords:** depression, lifestyle modification, primary care, randomized controlled trial (RCT), health promotion

## Abstract

**Background:**

Depression symptoms are prevalent in the general population, and their onset and continuation may be related to biological and psychosocial factors, many of which are related to lifestyle aspects. Health promotion and lifestyle modification programmes (LMPs) may be effective on reducing the symptoms. The objective of this study was to analyse the clinical effectiveness of a LMP and a LMP plus Information and Communication Technologies, when compared to Treatment as Usual (TAU) over 6 months. The interventions were offered as an adjuvant treatment delivered in Primary Healthcare Centers (PHCs) for people with depression symptoms.

**Methods:**

We conducted an open-label, multicentre, pragmatic, randomized clinical trial. Participants were recruited from several PHCs. Those participants visiting general practitioner for any reason, who also met the inclusion criteria (scoring 10 to 30 points on the Beck II Self-Applied Depression Inventory) were invited to take part in the study. TAU+LMP consisted of six weekly 90-min group sessions focused on improving lifestyle. TAU+LMP + ICTs replicated the TAU+LMP format, plus the addition of a wearable smartwatch to measure daily minutes walked and sleep patterns. A total of 188 participants consented to participate in the study and were randomized. We used linear mixed models, with a random intercept and an unstructured covariance to evaluate the impact of the interventions compared to TAU.

**Results:**

Both interventions showed a statistically significant reduction on depressive symptoms compared to TAU (TAU+LMP vs. TAU slope difference, b = −3.38, 95% CI= [−5.286, −1.474] *p* = 0.001 and TAU+LMP+ICTs vs. TAU slope difference, b = −4.05, 95% CI = [−5.919, −2.197], p < 0.001). These reductions imply a moderate effect size. In the TAU+LMP+ICTs there was a significant increase regarding minutes walking per week (b = 99.77) and adherence to Mediterranean diet (b = 0.702). In the TAU+LMP there was a significant decrease regarding bad sleep quality (b = −1.24).

**Conclusion:**

TAU+LMPs administered in PHCs to people experiencing depression symptoms were effective on reducing these symptoms compared to TAU. They also have a positive impact on changing several lifestyle factors. These findings indicate that these interventions can be promising strategies for PHCs.

## Introduction

It is estimated that 280 million people of all ages are currently experiencing depression symptoms and their impact (1). Due to its high prevalence in primary care settings (2), its treatment at this level of care is recommended (3, 4). One of the goals of primary care interventions is about educating people about healthy lifestyle habits (5). Lifestyle Modification Programmes (LMPs) can prevent the development of depression and are considered a successful treatment option (6–9). Regarding specific lifestyle factors, regular leisure-time exercise of any intensity has been shown to improve mental health and prevent depression (10, 11). Moreover, sleep disturbance is not only a manifestation of depression but can also be considered a prodromal symptom, therefore, its identification and treatment needs to be prioritized before, during and after experiencing depression (12, 13).

Spain has long been associated with the Mediterranean diet, which is regarded as one of the world's healthiest dietary patterns (14). This dietary pattern may be a safe and low-cost measure for depression prevention (15).

Additionally, facilitators of adherence to interventions, such as the use of Information and Communication Technologies (ICTs), should be considered in LMPs (16). In previous studies, the practice of monitoring behaviors in daily life has been useful to promote lifestyle changes in depressed people in primary care (17, 18). More specifically, wearable devices have been proven to be feasible and acceptable for use among overweight people with severe mental illness (19). These devices allow for monitoring behaviors in real-time in an unobtrusive way, enabling people to monitor and change their own activity (20).

Therefore, specifically, and as a novelty of this study, we combined and promoted several healthy lifestyles together (physical activity, sleep patterns and diet) in a face-to-face group format at the primary healthcare level with a longitudinal follow-up at 6 months in order to evaluate the effectiveness of the interventions over time.

The main objective is to analyse the clinical effectiveness of a TAU+LMP and an TAU+LMP with ICTs, when compared to Treatment as Usual (TAU) over 6 months, delivered in the context of in Primary Healthcare Centers (PHCs) as an adjuvant treatment for people experiencing depression symptoms. The second objective is to analyse if both interventions are similarly effective in improving the results of the lifestyle variables when compared to TAU.

## Methods and analysis

### Study design

An open-label, multicentre, pragmatic, randomized clinical trial (RCT) in three parallel groups was carried out: TAU as a control group, and TAU+LMP and TAU+LMP+ICTs as intervention groups in several PHCs.

### Sample size

To estimate the sample needed for this study, a Spanish study conducted with primary care patients with depression was considered as a proxy reference (18). Serrano-Ripoll et al. (18) reported an average score in BDI-II (21) at baseline of 24.5 points (SD 7.84). Following Button et al. (22) recommendation of considering a 17.5% reduction in the BDI-II as clinically relevant, we determined that a decrease of at least 4.28 points would be clinically significant and beneficial for people in Spain. Accepting a risk of 0.05 and a risk of 0.20 in a bilateral contrast, each treatment group required 35 participants. A final sample size of 42 people per each group was considered, with consideration of having a possible 20% withdrawal rate. The total sample size required was 126.

### Recruitment and participants

Participants were chosen from among those who visited a general practitioner (GP) at one of the participating PHCs for any reason and who also met the inclusion criteria described below. The recruiting time was 7 months (starting in April 2020 and finishing in October 2020). By the end of the study, 188 patients from PHCs in two locations in Spain (Zaragoza and Mallorca) with subclinical, mild or moderate depression (scoring 10 to 30 points on the BDI-II) (21) were recruited for the study. Further details about the inclusion and exclusion criteria are available in the published protocol (23).

A computer-generated random number (24) administered by an independent researcher was used to allocate participants. All the study centers randomized patients to all conditions.

### Intervention development and evaluation

All participants received a general medical care from their GPs, which means that they received the care they usually get in PHC, which typically does not mean care from clinicians specialized in delivering mental health care (25). In Spain general medical care could be usual antidepressant treatment with psychological advice and / or psychotropic drugs by the GP (26).

Those allocated to TAU+LMP received 90-min session per week for 6 weeks conducted by an expert psychologist, which were also supplemented with PowerPoint presentations. The following topics were covered: 1) Psychoeducation on depression; 2) Behavior activation; 3) Sleep hygiene habits and careful exposure to sunlight; 4) Physical activity; 5) Adherence to the Mediterranean diet; and 6) Summary of previous sessions. TAU+LMP+ICTs replicated the TAU+LMP format, plus the addition that participants were given a wearable smartwatch and instructed to wear it to measure daily minutes walked and sleep patterns. Those participants not assigned to either of the two interventions were considering as part of TAU group (25).

A blinded research assistant gathered patient data by administering questionnaires at baseline (T0), immediately after the intervention (T1), and at six-month follow-up session (T2).

### Outcomes and measures

Data about gender, age, marital status, level of education, occupation and economic level were collected. Chronic comorbidities with prevalences >5% were also considered (arrhythmias, heart failure, ischemic cardiopathy, dyslipidemia, obesity, excess weight, vein and artery disease, cerebrovascular disease, diabetes, chronic bronchitis, chronic obstructive pulmonary disease (COPD), asthma, chronic kidney disease, hypo and hyperthyroidism, tobacco use, alcoholism, insomnia, attempted suicide, anemia, neoplasia, dementia, deafness, cataracts, glaucoma, arthrosis, osteoporosis, and back pain) (27).

The primary outcome was the severity of depressive symptoms, measured by the BDI-II. It consists of 21 questions, with higher scores indicating more severe depressive symptomatology (28). The internal consistency of the BDI-II in our sample was acceptable at baseline (α = 0.71).

#### Secondary outcomes

To analyse the effectiveness of the intervention in modifying lifestyles, physical activity was measured using the International Physical Activity Questionnaire-Short Form (IPAQ-SF) (29). It assesses the activity over the last seven days (30) and contains seven items. In our analysis, we use the minutes walking per week and the minutes seated per day.

Adherence to the Mediterranean diet was assessed using the 14-item Mediterranean Diet Adherence Screener (MEDAS), developed by the PREDIMED study group (31). It includes items related to food consumption and consumption habits. Higher scores indicate higher level of adherence (32).

Sleep quality and sleep patterns were measured using the Pittsburgh Sleep Quality Index (PSQI) (33), which consists of 19 questions about subjective sleep quality, sleep latency, sleep duration, habitual sleep efficiency, sleep disturbances, sleep medication use and daytime dysfunction over the previous month. Higher scores indicate worse sleep quality (34). The internal consistency of the PSQI in our sample was acceptable at baseline (α = 0.75).

### Ethics approval

Ethics approval was granted by the Research Ethics Committee of Aragón (CEICA, PI18/286) and the Research Ethics Committee of the Balearic Islands (IB3950/19 PI). The study was developed following the Helsinki Declaration. All of the subjects signed an informed consent form; their data were anonymized and were only used for the purposes of the study.

### Statistical analysis

Firstly, a descriptive analysis (frequencies for categorical variables; means and standard deviation for continuous variables) and a univariate analysis (one-way ANOVA for age, BDI-II, IPAQ-SF, PSQI, and MEDAS, and Chi-Square test for the remaining variables) were used to examine the data and tested whether there were baseline differences between groups after randomization. Secondly, to answer the main objective – whether there were differences between treatment groups regarding their effectiveness in reducing depression—we used Linear Mixed-Effects Models (LMEMs) (35). We specified a model with a random intercept and unstructured covariance. The parameter of interest was the interaction effect of treatment and time in a model that also included age as a covariate because it was the only baseline variable that was significantly different between groups. Cohen's d (*d*) is calculated from the estimated mean values of BDI-II and its standard deviations (SD) at baseline (36).

Moreover, to answer the second question—whether there were differences between treatment groups with respect to the improvement of lifestyle variables—we used LMEMs with the same previous components.

The statistical analysis was carried out per intention-to-treat analysis (ITT) (i.e., all participants who were randomized were included in the statistical analysis and were analyzed according to the group to which they were originally assigned) (37). The results from the trial were presented as a regression coefficient for predicting change in primary and secondary outcomes with 95% confidence intervals. LMEMs were tested against a Bonferroni-adjusted alpha level of 0.01 (0.05/5) (38). A statistical analysis was performed using the SPSS software (version 25.0) (39).

## Results

**Figure 1:**
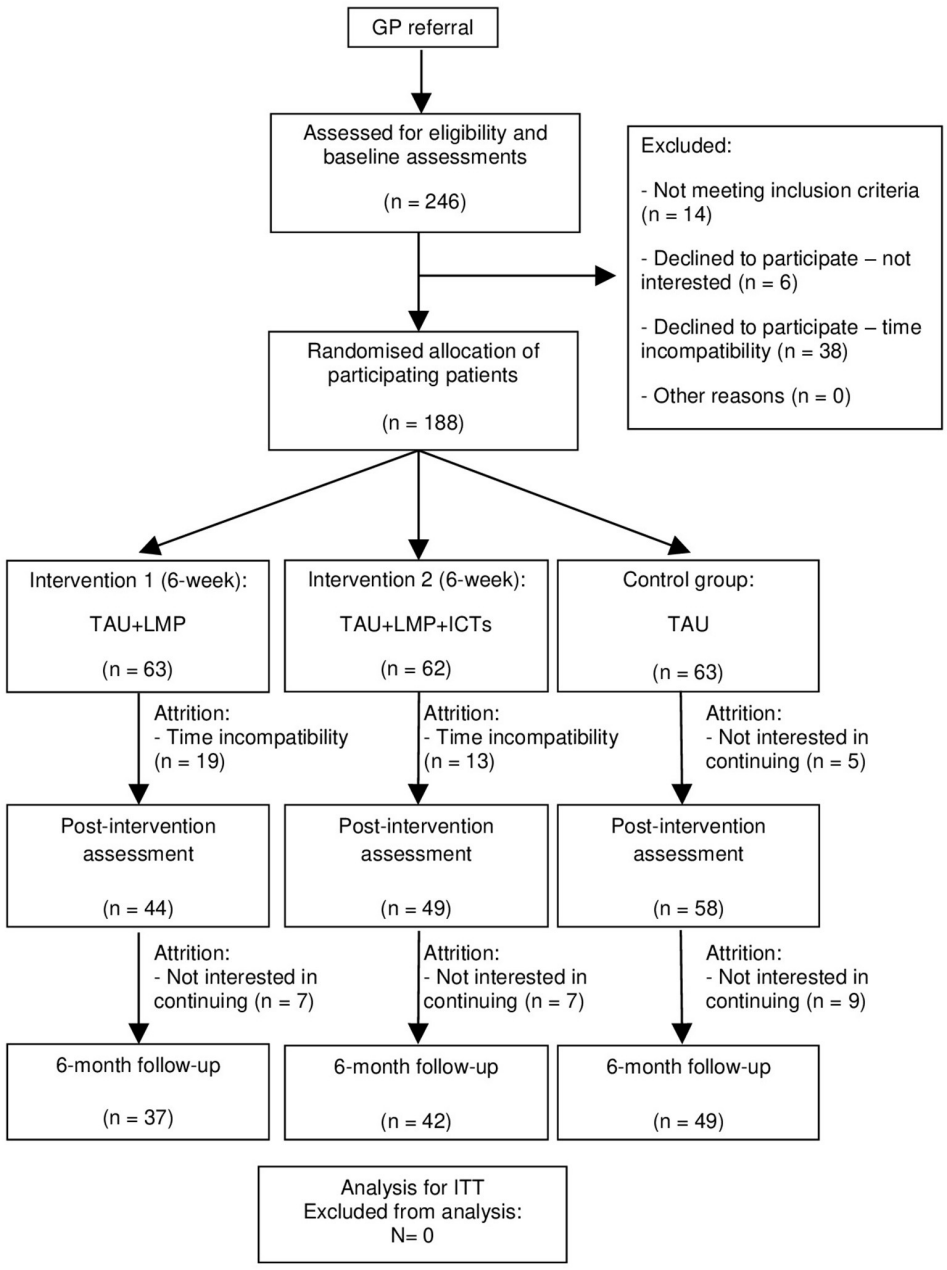
Flowchart of the study: randomization, sampling and monitoring of patients. GP, General Practitioner; TAU, Treatment as Usual; LMP, Lifestyle Modification Programme; ICTs, Information and Communication Technologies; ITT, Intention-to-treat.

A total of 246 participants were evaluated for eligibility, with 14 of them failing to meet the inclusion criteria, 6 declining to participate because they were not interested, and 38 declining to participate because they had time incompatibility. Of the 246 initial participants, 58 (23.58%) did not participate. Finally, 188 participants were included (Figure 1).

Firstly, the descriptive analysis showed that of the 188 participants, 162 were female and 26 were male, and all participants were between 20 to 83 years old (mean age = 53.32, SD = 13.07). The univariate analysis subsequently revealed significant differences between the groups (p = 0.014) regarding age, specifically between the TAU and TAU+LMP+ICTs groups (p = 0.018), with the TAU+LMP+ICT participants being older. However, no significant differences were found between the groups in the other variables (Table 1).

**Table 1:** Sociodemographic and clinical characteristics of the sample.

**Variables**	**Total (*****n*** = **188)**	**TAU (*****n*** = **63)**	**TAU**+**LMP (*****n*** = **63)**	**TAU**+**LMP**+**ICTs (*****n*** = **62)**	***p***
Age, *M* (*SD*)	53.32 (13.07)	49.54 (13.50)	54.35 (12.97)	56.11 (11.99)	0.014
Gender, *female n (%)*	162 (86.2)	52 (82.5)	54 (85.7)	56 (90.3)	0.448
Education
None or primary, *n (%)*	72 (38.3)	21 (33.3)	22 (34.9)	29 (46.8)	0.241
Secondary or tertiary, *n (%)*	116 (61.7)	42 (66.7)	41 (65.1)	33 (53.2)	
Occupation
Working, *n (%)*	53 (28.2)	23 (36.5)	17 (27)	13 (21)	0.150
Not working, *n (%)*	135 (71.8)	40 (63.5)	46 (73)	49 (79)	
Marital status
With a partner, *n (%)*	105 (54.4)	32 (50.8)	34 (54)	37 (59.7)	0.600
Without a partner, *n (%)*	88 (45.6)	31 (49.2)	29 (46)	25 (40.3)	
Economic level
< IMW to 2 IMW, *n (%)*	164 (87.2)	57 (90.5)	51 (81)	56 (90.3)	0.187
>2 IMW, *n (%)*	24 (12.8)	6 (9.5)	12 (19)	6 (9.7)	
Taking antidepressants, *yes n (%)*	132 (71.3)	45 (71.4)	46 (73)	43 (69.4)	0.776
N° of chronic comorbidities, *M* (*SD*)	4.51 (3.95)	4.03 (4.02)	4.41 (3.62)	5.09 (4.18)	0.314
BDI-II, *M* (*SD*)	24.90 (5.11)	24.13 (5.05)	25.00 (4.94)	25.58 (5.29)	0.278
Number of sessions attended^*^, *M* (*SD*)	4.98 (1.09)	-	5.07 (1.02)	4.90 (1.15)	0.451
IPAQ-SF-Walking, *M* (*SD*)	206.46 (273.95)	208.25 (324.92)	233.25 (261.87)	177.42 (226.87)	0.524
IPAQ-SF-Sedentarism, *M* (*SD*)	289.97 (186.24)	256.75 (212.23)	306.67 (180.20)	306.77 (160.72)	0.222
PSQI, *M* (*SD*)	11.66 (4.64)	11.57 (4.91)	12.11 (4.34)	11.29 (4.69)	0.605
MEDAS, *M* (*SD*)	6.47 (1.86)	6.41 (1.81)	6.54 (2.08)	6.45 (1.70)	0.927

*Significant differences (p ≤ 0.05) are highlighted in bold*.

* *Only patients in the intervention group who did not drop out were included. IMW, Interprofessional Minimum Wage. one-way ANOVA for age, n° of chronic comorbidities, BDI-II, IPAQ-SF, PSQI, and MEDAS, and Chi-Square test for the remaining variables. BDI-II, Beck II Self-Applied Depression Inventory; IPAQ-SF, Physical Activity Questionnaire-Short Form; PSQI, Pittsburgh Sleep Quality Index; MEDAS, Mediterranean Diet Adherence Screener; TAU, Treatment as Usual; LMP, Lifestyle Modification Programme; ICTs, Information and Communication Technologies*.

Considering the raw scores of both intervention groups, there was a decrease in BDI-II at 6 months compared to baseline levels (TAU+LMP mean difference = −5.48, SD = 9.50 and TAU+LMP+ICTs mean difference = −7.71, SD = 11.52). Also, an increase in IPAQ–SF–Walking (TAU+LMP mean difference = 123.56, SD = 251.74 and TAU+LMP+ICTs mean difference = 189.88, SD = 350.99), a decrease in IPAQ–SF–Sedentarism (TAU+LMP mean difference = −2.56, SD = 167.48 and TAU+LMP+ICTs mean difference = −32.62, SD = 189.61), a decrease in PSQI (TAU+LMP mean difference = −3.21, SD = 4.36 and TAU+LMP+ICTs mean difference = −1.52, SD = 5.13) and a decrease and an increase in MEDAS (TAU+LMP mean difference = −0.21, SD = 2.15 and TAU+LMP+ICTs mean difference =0.92, SD = 1.64) (Table 2).

**Table 2:** Outcome variables of each group in each measurement.

**Variables**	**TAU**	**TAU**+**LMP**	**TAU**+**LMP**+**ICTs**
BDI-II, *M* (*SD*)			
T0	24.13 (5.05)	25.00 (4.94)	25.58 (5.29)
T1	27.45 (9.08)	18.16 (8.53)	19.94 (8.08)
T2	24.00 (12.72)	18.49 (9.95)	17.69 (11.79)
T1-T0	3.29 (7.55)	−6.43 (7.77)	−5.59 (6.74)
T2-T0	−0.12 (12.06)	−5.48 (9.50)	−7.71 (11.52)
IPAQ-SF-walking, *M* (*SD*)			
T0	208.25 (324.92)	233.25 (261.87)	177.42 (226.87)
T1	177.93 (346.64)	380.56 (371.98)	367.34 (430.34)
T2	211.12 (279.09)	368.43 (338.27)	373.33 (351.61)
T1-T0	−13.96 (410.85)	145.34 (250.76)	205.30 (432.34)
T2-T0	5.51 (270.41)	123.56 (251.74)	189.88 (350.99)
IPAQ-SF-sedentarism, *M* (*SD*)			
T0	256.75 (212.23)	306.67 (180.20)	306.77 (160.72)
T1	280.69 (210.17)	240.11 (160.67)	248.57 (150.99)
T2	302.45 (188.73)	277.97 (172.68)	261.67 (184.03)
T1-T0	13.96 (223.10)	−51.70 (135.25)	−52.04 (149.22)
T2-T0	49.49 (211.65)	−2.56 (167.48)	−32.62 (189.61)
PSQI, *M* (*SD*)			
T0	11.57 (4.91)	12.11 (4.34)	11.29 (4.69)
T1	12.29 (3.95)	9.25 (4.12)	10.65 (4.91)
T2	10.59 (5.74)	8.70 (4.20)	9.85 (5.06)
T1-T0	0.51 (3.79)	−2.68 (3.77)	−0.73 (3.25)
T2-T0	−1.32 (5.11)	−3.21 (4.36)	−1.52 (5.13)
MEDAS, *M* (*SD*)			
T0	6.41 (1.81)	6.54 (2.08)	6.45 (1.70)
T1	5.98 (2.26)	7.18 (1.83)	7.20 (1.67)
T2	6.20 (1.98)	6.78 (1.73)	7.69 (1.52)
T1-T0	−0.41 (1.69)	0.41 (2.29)	0.61 (1.60)
T2-T0	−0.30 (1.89)	−0.21 (2.15)	0.92 (1.64)

*BDI-II, Beck II Self-Applied Depression Inventory; IPAQ-SF, Physical Activity Questionnaire-Short Form; PSQI, Pittsburgh Sleep Quality Index; MEDAS, Mediterranean Diet Adherence Screener; TAU, Treatment as Usual; LMP, Lifestyle Modification Programme; ICTs, Information and Communication Technologies; T0, Baseline assessment; T1, Post-intervention assessment; T2, Six-month follow-up*.

Secondly, the LMEM evidenced that both interventions could be clinically effective compared to TAU, as there was a significant interaction effect for both treatments and time on BDI–II (TAU+LMP vs. TAU slope difference: *b* = −3.38, 95% CI= [−5.286, −1.474] p =0.001; and TAU+LMP+ICTs vs. TAU slope difference: *b* = −4.06, 95% CI= [−5.919, −2.197], p <0.001) (Table 3). That reduction in BDI–II implies a moderate effect size in both TAU+LMP and TAU+LMP+ICTs groups (*d* =0.671 and *d* =0.779, respectively).

**Table 3:** Estimates of Fixed Effects in BDI-II.

**Parameter**	**Estimate**	**95% CI for estimated**	***SE***	**t**	***p***
Intercept	24.948	[22.947, 26.949]	1.017	24.524	<0.001
Time	0.118	[−1.158, 1.396]	0.649	0.183	0.855
Age	−0.050	[−0.127,0.026]	0.038	−1.291	0.198
TAU+LMP+ICTs	0.307	[−2.556, 3.171]	1.456	0.211	0.833
TAU+LMP	−0.749	[−3.588, 2.090]	1.443	−0.519	0.604
TAU+LMP+ ICTs × Time	−4.058	[−5.919, −2.197]	0.946	−4.289	<0.001
TAU+LMP × Time	−3.380	[−5.286, −1.474]	0.968	−3.489	0.001

*Significant differences (p ≤ 0.01) are highlighted in bold. CI, confidence interval; TAU, Treatment as Usual; LMP, Lifestyle Modification Programme; ICTs, Information and Communication Technologies*.

Moreover, LMEMs showed that the variables that measure lifestyle (IPAQ–SF–Walking, IPAQ–SF–Sedentarism, PSQI and MEDAS) changed differently when comparing TAU to the intervention group. Specifically, regarding IPAQ–SF–Walking, there was a significant increase in the TAU+LMP+ICTs group (TAU+LMP+ICTs vs. TAU slope difference: b = 99.778, 95% CI= [30.530, 169.026], *p* = 0.005) (Supplementary Table 1). That increase in IPAQ-SF-Walking implies a small effect size in the TAU+LMP+ICTs group (*d* = 0.310). Regarding IPAQ-SF-Sedentarism, there were no significant changes in any group (Supplementary Table 2). Regarding PSQI, there was a significant reduction in the TAU+LMP group (TAU+LMP vs. TAU slope difference: b = −1.240, 95% CI= [−2.126, −0.354], *p* = 0.006) (Supplementary Table 3). That decrease in PSQI implies a small effect size in the TAU+LMP group (*d* = 0.268). Finally, regarding MEDAS, there was a significant increase in the TAU+LMP+ICTs group (TAU+LMP+ICTs vs. TAU slope difference: b = 0.702, 95% CI= [.337, 1.066], *p* < 0.001) (Supplementary Table 4). That increase in MEDAS implies a small effect size in the TAU+LMP+ICTs group (*d* = 0.040).

## Discussion

The findings of this study indicate that over 6 months, TAU+LMPs were effective in decreasing depressive symptoms. Also, TAU+LMPs helped in the adoption of several healthier lifestyle behaviors when compared to TAU.

The findings of this study are consistent with other Spanish RCTs on psychoeducational group interventions delivered by PHC nurses for people with depression and physical comorbidity (40, 41). Furthermore, they are also consistent with a multidisciplinary online programmes that integrates evidence-based tactics from the fields of lifestyle medicine (42), as well as with a recent pilot RCT about a group-based lifestyle medicine for depression (43).

Recent meta-analyses of RCTs concluded that multi-component LMPs (with three lifestyle factors such as physical activity, nutritional advice, and sleep management) appeared to be effective in mitigating depressive symptoms (44, 45). A recent Spanish longitudinal cohort study (46), a cross-sectional study about health conditions, lifestyle factors and depression (47) and a recent meta-analysis of observational studies (48) have all shown that healthy lifestyles are associated with a reduced risk of depressive symptoms.

Regarding changes on lifestyle, reflecting a long-lasting effect of the interventions, people receiving the TAU+LMP+ICTs group significantly increased their total weekly minutes of walking (approximately, 1 h and three quarters) when compared to TAU. Participants following the TAU+LMP also increased their minutes of walking (approximately 1 h), but those results only showed a certain trend toward significance. A systematic review and a meta-analysis analyzing RCTs about the treatment effect of exercise on depression (49, 50) concluded that physical exercise is an effective intervention for depression. Moreover, evidence suggests that it is not only necessary to be physically active but also to limit the number of hours spent being sedentary (51). In the same line, in a cross-sectional study with primary care patients, depression symptoms were associated with physical inactivity (52). Participants from the TAU+LMP+ICTs group reduced their total daily minutes seated (~45 min) almost significantly. This lack of relationship found between sedentary lifestyle and depression may be due to the fact that what the participants did while sitting was not controlled. Therefore, time spent being sedentary could have been used doing pleasant leisure activities (i.e., watching TV, reading or using the computer) (53).

An RCT associated physical activity with elevated mood and with a significant reduction in the severity of insomnia symptoms (54). In our study, there was a significant reduction in bad sleep quality among the participants from the TAU+LMP. In a recent cross-sectional study, inadequate sleep was associated with most health disabilities and major depression (55). Furthermore, a meta-analysis concluded that a lack of good sleep quality is significantly associated with depression in older adults (56), and another meta-analysis stated that certain sleep disorders (nightmares and insomnia) increase the risk of suicidal behavior in depressed patients (57).

A recent cross-sectional study observed positive associations between a healthy diet and sleep with mental health (58). Participants from the TAU+LMP+ICTs group significantly increased their adherence to the Mediterranean diet. A recent RCT determined that adherence to the Mediterranean diet was related to fewer depressive symptoms (59). A meta-analysis of RCTs determined that dietary interventions significantly reduced depressive symptoms (60). Moreover, meta-analyses of observational studies indicated that adults following a healthy dietary pattern have fewer depressive symptoms and a lower risk of developing depressive symptoms (61). In particular, adhering to the Mediterranean diet appeared to reduce the risk of depression (62).

The differences found between both interventions (TAU+LMP and TAU+LMP+ICTs) could be due to the use of the wearable smartwatch for monitoring. We have found the following advantages of its use. Firstly, the patients from the TAU+LMP+ICTs group had a more remarkable reduction in their depression and, as previously stated, this reduction of depression might be clinically relevant. Secondly, these patients had significantly increased the minutes of walking per week and they also increased their adherence to the Mediterranean diet. However, participants from this group did not have a significant improvement in their sleep quality, whereas the participants from the TAU+LMP improved their sleep. These results may have been influenced by the individual use of the smartwatch. Most of the patients from the TAU+LMP+ICTs group were very excited about using this device, however, most of them did not wear it during the night. As such, feedback about their sleep was not available. This underuse of the smartwatch could have been due to the patients' acceptance of technologies (63).

Regarding the limitations and strengths of the study, the first strength was the study's design; a pragmatic RCT with sample homogeneity between groups. As the study was developed in primary care conditions, the research results are easily transferable to practice. Another advantage is that since the randomization was blind, the evaluations and the statistical analysis provided greater validity to the results. Furthermore, and as a new characteristic, numerous aspects of healthy lifestyles were considered together and no adverse effects from the interventions were reported. In this regard, the group intervention format offered social support, a sense of belonging and the opportunity to share common difficulties (64). Finally, the participant profile was similar to regular PHC patients.

One limitation was the overlap with COVID-19 since participants found it difficult to properly implement the lifestyle guidelines during this time (65). Despite session attendance being high (5 out of the 6 sessions), another issue was the dropout rate, which was mostly due to time incompatibility or a lack of interest in answering the questionnaires during the follow-up. Furthermore, the sample was predominantly female and, as such, no analysis by gender could be performed. Finally, due to the nature of the intervention, both the psychologist who led the sessions and the participants were aware of the assigned intervention during the RCT.

Future trials with larger sample sizes could plan for subgroup analyses. For example, analyzing the effectiveness of an TAU+LMP for the different severities of depression (i.e., subclinical, mild, moderate and even severe). Moreover, recruiting more men may be beneficial to be able to make gender comparisons. Furthermore, adherence strategies (i.e., sending text messages) (66) may be considered in future RCTs. In addition, qualitative studies should be carried out to investigate the specific causes of dropout. Regarding the use of ICTs, more studies are needed to determine how to improve adherence and compliance rates so that people can wear wearable devices continuously for 24 h (20).

## Conclusion

TAU+LMPs administered in PHCs to people suffering from mainly moderate depression were effective in reducing depressive symptomatology comparing to TAU. The use of ICTs resulted in a greater improvement in depression and in several lifestyle factors (weekly minutes of walking and adherence to the Mediterranean diet). More research is needed to enhance adherence. These promising programmes could be easily implemented in PHCs.

## Data availability statement

The raw data supporting the conclusions of this article will be made available by the authors, without undue reservation.

## Ethics statement

The studies involving human participants were reviewed and approved by Research Ethics Committee of Aragón (CEICA, PI18/286). The patients/participants provided their written informed consent to participate in this study.

## Author contributions

All authors made a significant contribution to the work reported, whether that is in the conception, study design, execution, acquisition of data, analysis and interpretation, or in all these areas, took part in drafting, revising or critically reviewing the article, gave final approval of the version to be published, have agreed on the journal to which the article has been submitted, and agreed to be accountable for all aspects of the work.

## Funding

This work was supported by Carlos III Health Institute grant number PI18/01336. The funders have no role in study design, data collection and analysis, decision to publish or manuscript preparation. The funding organization will audit trial conduct once a year.

## Conflict of interest

The authors declare that the research was conducted in the absence of any commercial or financial relationships that could be construed as a potential conflict of interest.

## Publisher's note

All claims expressed in this article are solely those of the authors and do not necessarily represent those of their affiliated organizations, or those of the publisher, the editors and the reviewers. Any product that may be evaluated in this article, or claim that may be made by its manufacturer, is not guaranteed or endorsed by the publisher.
