# Supplementary material for: Effectiveness of a lifestyle modification programme in the treatment of depression symptoms in primary care
Source: Front Med (Lausanne). 2022 Jul 26;9:954644. doi: 10.3389/fmed.2022.954644 (PMC9361711; doi:10.3389/fmed.2022.954644)
Supplement: Supplementary file 1 [file Data_Sheet_1.docx]

**Supplementary**

**Supplementary Table 1. Estimates of Fixed Effects in IPAQ-Walking**

| Parameter | Estimate | 95% CI for estimated | *SE* | t | *p* |  |
| --- | --- | --- | --- | --- | --- | --- |
| Intercept | 196.860 | [119.613, 274.107] | 39.267 | 5.013 | < .001 |  |
| Time | 6.980 | [-40.497, 54.459] | 24.128 | .289 | .773 |  |
| Age | -.329 | [-3.341, 2.682] | 1.527 | -.216 | .829 |  |
| TAU+LMP+ICTs | 6.047 | [-104.523, 116.618] | 56.207 | .108 | .914 |  |
| TAU+LMP | 56.190 | [-53.383, 165.765] | 55.703 | 1.009 | .314 |  |
| TAU+LMP+ICTs × Time | **99.778** | **[30.530, 169.026]** | 35.195 | 2.835 | **.005** |  |
| TAU+LMP × Time | 61.001 | [-9.946, 131.949] | 36.063 | 1.692 | .092 |  |
| *Note*. Significant differences (p ≤ .01) are highlighted in bold. IPAQ, Physical Activity Questionnaire; CI, confidence interval; TAU, Treatment as Usual; LMP, Lifestyle Modification Programme; ICTs, Information and Communication Technologies. | | | | | |  |

**Supplementary Table 2. Estimates of Fixed Effects in IPAQ-Sedentarism**

| Parameter | Estimate | 95% CI for estimated | *SE* | *t* | *p* |
| --- | --- | --- | --- | --- | --- |
| Intercept | 254.096 | [210.366, 297.827] | 22.222 | 11.434 | < .001 |
| Time | 24.794 | [-.247, 49.835] | 12.724 | 1.948 | .052 |
| Age | -.201 | [-1.948, 1.545] | .885 | -.228 | .820 |
| TAU+LMP+ICTs | 43.470 | [-19.119, 106.059] | 31.805 | 1.367 | .173 |
| TAU+LMP | 40.656 | [-21.326, 102.640] | 31.499 | 1.291 | .198 |
| TAU+LMP+ICTs × Time | -44.318 | [-80.895, -7.740] | 18.588 | -2.384 | .018 |
| TAU+LMP × Time | -34.893 | [-72.417, 2.631] | 19.071 | -1.830 | .068 |
| *Note*. IPAQ, Physical Activity Questionnaire; CI, confidence interval; TAU, Treatment as Usual; LMP, Lifestyle Modification Programme; ICTs, Information and Communication Technologies. | | | | | |

**Supplementary Table 3. Estimates of Fixed Effects in PSQI**

| Parameter | Estimate | 95% CI for estimated | *SE* | t | *p* |  |
| --- | --- | --- | --- | --- | --- | --- |
| Intercept | 11.884 | [10.758, 13.010] | .572 | 20.775 | < .001 |  |
| Time | -.518 | [-1.108, .070] | .299 | -1.732 | .084 |  |
| Age | -.011 | [-.057, .034] | .023 | -.501 | .617 |  |
| TAU+LMP+ICTs | -.551 | [-2.163, 1.060] | .818 | -.674 | .501 |  |
| TAU+LMP | -.004 | [-1.599, 1.589] | .810 | -.006 | .995 |  |
| TAU+LMP+ICTs × Time | -.210 | [-1.072, .652] | .438 | -.479 | .632 |  |
| TAU+LMP × Time | **-1.240** | **[-2.126, -.354]** | .450 | -2.755 | **.006** |  |
| *Note*. Significant differences (p ≤ .01) are highlighted in bold. PSQI, Pittsburgh Sleep Quality Index; CI, confidence interval; TAU, Treatment as Usual; LMP, Lifestyle Modification Programme; ICTs, Information and Communication Technologies. | | | | | |  |

**Supplementary Table 4. Estimates of Fixed Effects in MEDAS**

| Parameter | Estimate | 95% CI for estimated | *SE* | t | *p* |
| --- | --- | --- | --- | --- | --- |
| Intercept | 6.441 | [5.999, 6.882] | .224 | 28.695 | < .001 |
| Time | -.159 | [-.408, .090] | .126 | -1.255 | .210 |
| Age | **.029** | **[.012, .047]** | .008 | 3.336 | **.001** |
| TAU+LMP+ICTs | -.034 | [-.666, .597] | .321 | -.108 | .914 |
| TAU+LMP | .191 | [-.434, .817] | .318 | .603 | .547 |
| TAU+LMP+ICTs * Time | **.702** | **[.337, 1.066]** | .185 | 3.793 | **< .001** |
| TAU+LMP * Time | .177 | [-.196, .551] | .189 | .934 | .351 |
| *Note*. Significant differences (p ≤ .01) are highlighted in bold. MEDAS, Mediterranean Diet Adherence Screener; CI, confidence interval; TAU, Treatment as Usual; LMP, Lifestyle Modification Programme; ICTs, Information and Communication Technologies. | | | | | |
